# Poor prognosis, hypomethylation, and immune infiltrates are associated with downregulation of INMT in head and neck squamous cell carcinoma

**DOI:** 10.3389/fgene.2022.917344

**Published:** 2022-09-15

**Authors:** Kun Cui, Xi Yao, Zhengbo Wei, Yujia yang, Xinli Liu, Zhongheng Huang, Huimin Huo, Jinping Tang, Ying Xie

**Affiliations:** ^1^ Guangxi Key Laboratory of High‐Incidence Tumor Prevention and Treatment, Guangxi Medical University, Ministry of Education, Nanning, Guangxi, China; ^2^ Life Sciences Institute of Guangxi Medical University, Nanning, Guangxi, China; ^3^ Department of Head and Neck Tumor Surgery, Affiliated Tumor Hospital of Guangxi Medical University, Nanning, China

**Keywords:** Indiolethylamine-N-methyltransferase, head and neck squamous cell carcinoma, low expression, poor prognosis, methylation, immune infiltration

## Abstract

**Background:** Indiolethylamine-N-methyltransferase (INMT) is a methyltransferase responsible for transferring methyl groups from methyl donor SAM to its substrate. S-adenosyl-l-methionine (SAM), obtained from the methionine cycle, is a naturally occurring sulfonium compound that is vital to cellular metabolism. The expression of INMT is down-regulated in many tumorous tissues, and it may contribute to tumor invasion and metastasis. Nevertheless, the expression of INMT and its relationship to methylation and immune infiltrates in head and neck squamous cell carcinoma (HNSC) remains a mystery. Thus, we evaluated expression, clinicopathological features, prognosis, several critical pathways, DNA methylation, and immune cell infiltration for the first time.

**Methods:** Analysis of the clinicopathological characteristics of INMT expression, several tumor-related bioinformatics databases were utilized. In addition, the role of INMT expression was analyzed for prognosis. Several INMT-related pathways were enriched on the LinkedOmics website. In addition, we have analyzed the methylation of INMT in HNSC in detail by using several methylation databases. Lastly, the relationship between INMT gene expression and immune infiltration was analyzed with ssGSEA, Timer, and TISIDB.

**Results:** In HNSC, mRNA and protein levels were significantly lower than in normal tissues. The low expression of INMT was statistically associated with T stage, histological grade, gender, smoking history, and alcohol consumption. HNSC patients with low INMT expression have a poorer OS (overall survival) compared to those with high levels of expression. In addition, the multivariate analysis revealed INMT expression to be a remarkable independent predictor of prognosis in HNSC patients. An analysis of gene enrichment showed that several pathways were enriched in INMT, including the Ras signaling pathway, the cGMP-PKG signaling pathway, and others. Moreover, methylation patterns of INMT detected in a variety of methylation databases are closely associated with mRNA expression and prognosis. Finally, INMT was significantly correlated with immune infiltration levels.

**Conclusion:** HNSC with low levels of INMT exhibits poor survival, hypomethylation, and immune infiltration. For HNSC, this study presented evidence that INMT is both a biomarker of poor prognosis and a target of immunotherapy.

## Introduction

Around 700,000 cases of head and neck squamous cell carcinoma (HNSC) occur worldwide annually (Siegel et al., 2020). The 5-year overall survival (OS) for HNSC patients has remained at 60%, despite the advancing treatment (Chi et al., 2015). When determining the prognosis of patients with HNSC, the TNM classification system considers tumor size, location, and metastatic status. A treatment strategy is then developed (Lydiatt et al., 2017). The TNM system is not without its flaws, however, as patients with the same stage of cancer react to treatments differently (Budach and Tinhofer, 2019). As a result of HNSC’s high degree of heterogeneity, biomarkers must be stable, reliable, and broad-spectrum (O’Sullivan et al., 2013). As a consequence, HNSC requires useful therapeutic targets or identification of potential prognostic biomarkers.

DNA methylation is one of the most important epigenetic modifications (Baylin and Jones, 2016), the nucleic acid sequence does not change, and the gene expression can be inherited, playing key roles in the regulation of gene expression, genomic imprinting, X chromosome inactivation, and tumorigenesis (Jones and Baylin, 2007; Smith and Meissner, 2013). DNA methylation is catalyzed by a family of DNA methyltransferases (Dnmts) that transfer a methyl group from S-adenyl methionine (SAM) to the fifth carbon of a cytosine residue to form 5 mC (Moore et al., 2013). As an essential amino acid, methionine is found in the diet of mammals. It is converted to the principal cellular methyl donor, S-adenosylmethionine (SAM, also known as AdoMet), by the transfer of adenosine from ATP to the methionine sulfur. Methionine adenosyltransferase (MAT) is responsible for catalyzing this reaction (Kaiser, 2020). SAM is a naturally arising sulfonium compound that is essential for cellular metabolism. SAM has been shown to slow the progression of several types of human tumors over the last few decades (Ansorena et al., 2002; Lu and Mato, 2008; Martinez-Lopez et al., 2008). Indolethylamine-N-methyltransferase (INMT) is a methyltransferase that transfers one or more methyl groups from the methyl donor S-adenosyl-l-methionine (SAM) to the substrate (Axelrod, 1962; Herman et al., 1985; Thompson and Weinshilboum, 1998). It is, therefore, demonstrated that INMT contributes to the detoxification of selenium compounds and that it is involved in the regulation of the tryptophan metabolic pathway (Kuehnelt et al., 2015). Researchers found that INMT levels were lower in lung, meningioma, and prostate cancers (Kopantzev et al., 2008; Larkin et al., 2012; Schulten et al., 2016); however, the role of INMT in cancer is unclear.

The purpose of this article is to investigate the role of INMT in HNSC and its potential prognostic value. In the first step of the process, we gathered comprehensive gene expression data, clinical information, and prognostic information about HNSC patients from TCGA as well as other sources. The second step was to analyze protein expression, protein-protein interactions, and functional enrichment in HNSC using various related databases. Moreover, we used several methylation databases to analyze the methylation of INMT in HNSC in detail. Finally, the tissue microenvironment of tumor cells plays an important role in tumor development, which led us to explore the relationship between immune cells and INMT in the immune microenvironment of HNSC. Based on the results of this study, INMT may be utilized as either an indicator of prognosis or a therapeutic target for HNSC.

## Materials and methods

### Data acquisition and processing

Our study consists of extracting RNA-Seq expression data and clinical information associated with INMT in HNSC from the TCGA official website (Tomczak et al., 2015). So, 502 HNSC samples, as well as 44 adjacent normal tissue samples, were retained for analysis. To further analyze gene expression data obtained through RNA-Seq, the FPKM generated workflow data was converted to TPM format, and a log2 conversion was performed. Genomic information on INMT was also collected from selected samples, including TNM stage, clinical stage, histological grade, age, sex, smoking history, drinker, and radiation therapy. The mRNA expression data were presented as mean ± standard deviation. A Pearson correlation analysis was also utilized to determine the association between INMT expression levels and immune checkpoint gene expression. Since the research was conducted using data obtained from TCGA, it was not necessary to obtain Ethics Committee approval for this research. As a final step, gene expression profiles of GSE30784 were acquired from the Gene Expression Omnibus (GEO) database (Edgar et al., 2002) to further verify that INMT was downregulated in HNSC tissues.

### Analyze on the TIMER website

TIMER (Li et al., 2017) is a web server that performs analyses of gene expression and immunological cells that infiltrate tumors of a variety of cancer types. Based on analyses of TIMER, we assessed whether INMT is differentially expressed in a variety of tumor types as compared to normal tissues. As part of our study, we explored the association between INMT and six immune infiltrating cells of the tumor as well as 16 molecular markers of immune cells.

### Kaplan-Meier Plotter database analysis

The Kaplan-Meier plotter (Nagy et al., 2018) is an open, intuitive online tool that can be used to perform prognostic analysis in multiple cancer tissues. Based on the Kaplan-Meier plotter website, a link between clinical outcomes and INMT expression in HNSC was first assessed. The level of INMT expression within related immune cell subsets was then utilized for prognostic analysis. A hazard ratio (HR) based on 95% confidence intervals (CIs) was calculated along with the log-rank *p*-value.

### Indiolethylamine-N-methyltransferase protein expression, functional enrichment analysis, and protein-protein interaction networks

UALCAN’s website makes it easy to analyze publicly available cancer data, such as protein expression, by providing easy-to-use tools such as CPTAC (Edwards et al., 2015; Chandrashekar et al., 2017). Using UALCAN, we examine the expression of INMT proteins throughout CPTAC.

Using the LinkFinder module on the LinkedOmics website (Vasaikar et al., 2018), the differentially expressed genes associated with INMT were analyzed from the TCGA HNSC dataset, and Pearson correlation coefficients were utilized to determine the correlation between the results, which were represented in a volcano plot and heat map, respectively. An analysis of Gene Ontology (biological processes, cellular components, and molecular functions) and Kyoto Encyclopedia of Genes and Genomes (KEGG) pathways using Gene Set Enrichment Analysis (GSEA) is performed by Link Interpreter. Furthermore, we employed the GEPIA database (Tang et al., 2017) to visualize heat maps showing the top 50 genes positively and negatively associated with INMT, respectively.

Protein-protein interactions (PPI) of INMT-binding proteins were analyzed on the STRING database (Szklarczyk et al., 2011) with parameters like the meaning of edges of the network (“evidence”), active sources of interaction (“experiments”), the minimum needed score for an interaction study [“Low confidence (0.150)”], and the maximum number of interactions to be calculated (“no more than 50 interactors”). Afterward, the information about 50 INMT-binding proteins with experimental evidence was identified from the interaction network. The intersection analysis of INMT-co-expressed genes and INMT-interacted genes was performed using an interactive Venn diagram viewer. Utilizing the Timer, we investigated the link between INMT expression and the common genes identified through intersection analysis.

### Methylation and expression analysis of Indiolethylamine-N-methyltransferase

Researchers have found that DNA methylation is a significant epigenetic mechanism capable of governing gene expression and influencing cancer cell behavior. UCSC Xena is a genome-based database (Goldman et al., 2020), and we used this database for the analysis of INMT methylation and expression. After that, we analyzed methylation levels of INMT on both HNSC and paracancerous normal tissues using UALCAN and DiseaseMeth version 2.0 (Xiong et al., 2017). Additionally, using MEXPRESS (Koch et al., 2015), INMT expression was correlated with DNA methylation status in our study. Finally, we performed a multivariate survival analysis using MethSurv (Modhukur et al., 2018) to determine the distribution of CpG islands.

### Immune infiltration analysis

In the study conducted by Bindea et al. (2013), the marker genes were extracted from 24 immune cells. To determine the amount of tumor-infiltrating immune cells, single-sample GSEA (ssGSEA) (Finotello and Trajanoski, 2018) was initially employed using HNSC mRNA TPM data from the TCGA.

Spearman correlation was applied to establish the correlation between INMT and these 24 types of immune cells. A database called TISIDB is available for analyzing tumor-immune cell interaction (Ru et al., 2019), and we also investigated the relation of INMT expression and methylation to the presence of tumor-infiltrating lymphocytes using this platform.

### Statistical analysis

We used the R (V 3.6.3) and R package ggplot2 to display the expression differences to conduct statistical analyses. Comparing HNSC tissues and adjacent normal tissues were accomplished using paired t-tests and Mann-Whitney *U*-tests. To investigate the relationship between clinicopathological features and INMT expression, Mann-Whitney *U*-test, Fisher’s test, Chi-Squared test, and logistic regression were employed. With the pROC package (V 1.17.0.1), INMT expression was assessed for diagnostic accuracy using ROC curves. To assess the effect of INMT on survival, Kaplan-Meier and log-rank tests were performed using the survminer package (V 0.4.9). To estimate the risk of death, we performed multivariate and univariate analyses using Cox proportional hazard models. In multivariate Cox regression analysis, variables with *p* < 0.15 in univariate Cox regression are included in the analysis. As a result of the Cox regression models, the independent prognostic factors acquired from the multivariate analysis were employed to develop nomograms, each predicting the probability of survival at 1-, 3-, and 5-years. Based upon the RMS package (V 6.2-0), we created nomograms containing important clinical characteristics as well as calibration plots. We calculated the discrimination of the nomogram utilizing a concordance index (C-index). Our study employed two-tailed statistical tests with a significance level of 0.05 or less.

## Results

### Abnormally low level of Indiolethylamine-N-methyltransferase expression in head and neck squamous cell carcinoma

**FIGURE 1:**
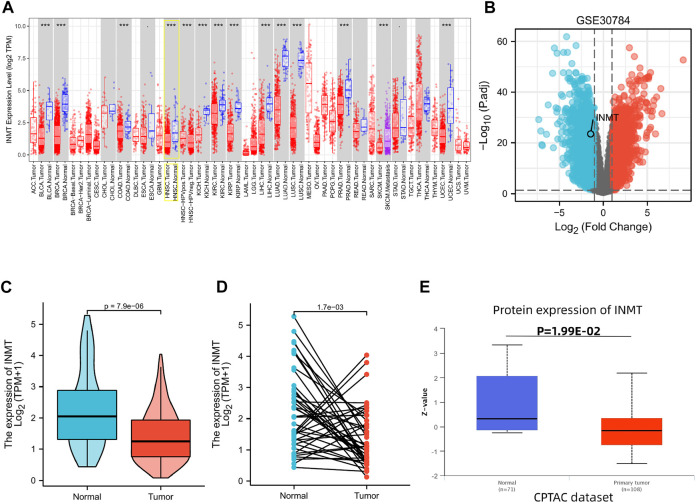
Variations in the expression levels of INMT between malignancies and the mRNA and protein expression levels in HNSC. **(A)** The expression level of INMT in multiple tumors derived from TCGA data in TIMER. Note: *p < 0.05, **p < 0.01, ***p < 0.001. **(B)** Volcano plots of the DEGs in GEO. **(C,D)** Differential levels of INMT mRNA expression in HNSC. **(E)** The expression level of INMT protein according to CPTAC.

Initially, we utilized the TIMER website to probe the mRNA expression of INMT. INMT mRNA expression was significantly lower in multiple human cancers, specifically in HNSC, in comparison with their respective normal tissues (Figure 1A). After that, we selected dataset GSE30784 (Chen et al., 2008) from the HNSC database to identify differentially expressed genes (DEGs). Comparing samples of 167 HNSC patients with low INMT with 62 samples of patients with high INMT. The analysis identified 3630 DEGs that covered 1898 upregulated genes and 1732 downregulated genes as statistically significant between the two cohorts (|Log2-fold change| > 1, adjusted *p*-value < 0.05). We still found that INMT was down-regulated in the differentially expressed genes identified (Figure 1B).

The INMT expression data from TCGA were further analyzed to demonstrate the mRNA and protein expression of INMT in HNSC. Unpaired data analysis illustrated that the mRNA expression of INMT was statistically lower in HNSC samples (*n* = 502) as compared with normal samples (*n* = 44) (Figure 1C, 1.411 ± 0.83 vs. 2.255 ± 1.26, *p* < 0.001). Additionally, an analysis of paired data indicated that the levels of mRNA expression of INMT in HNSC tissues (*n* = 43) were statistically lower than those in adjacent normal tissues (*n* = 43) (Figure 1D, 1.391 ± 0.944 vs. 2.263 ± 1.274, *p* = 0.002).

As a final step, we analyzed CPTAC with UALCAN to analyze the expression of INMT protein. It was found that the protein expression of INMT in HNSC was significantly lower than in normal tissues (Figure 1E, *p* < 0.05).

### Association with Indiolethylamine-N-methyltransferase expression and clinicopathological variables

**FIGURE 2:**
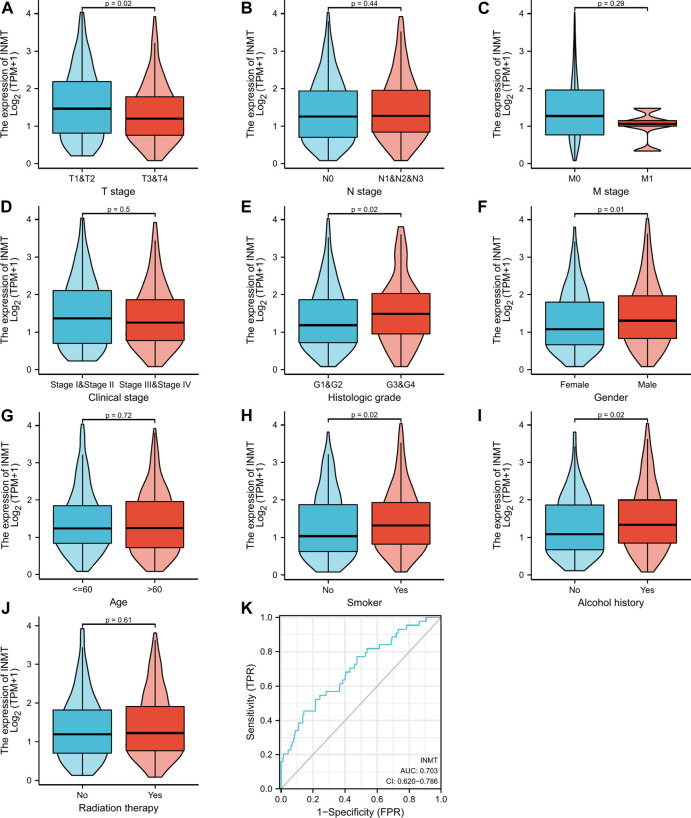
Clinical pathological characteristics correlated with INMT mRNA expression levels using the TCGA dataset. INMT mRNA expression was statistically related to T stage **(A)**, histologic grade **(E)**, gender **(F)**, smoke **(H)**, and alcohol history **(I)**. Nonetheless, no statistical association was discovered between the expression levels of INMT and N stage **(B)**, M stage **(C)**, clinical stage **(D)**, age **(G)**, and radiation therapy **(J)**. ROC analysis of INMT in HNSC **(K)**.

Firstly, a Mann-Whitney *U*-test was conducted to develop an understanding of the link between the INMT expression and clinical-pathological characteristics of HNSC tissues. According to Figures 2A–K, decreased INMT was remarkably correlated with T stage (*p* = 0.02), histological grade (*p* = 0.02), gender (*p* = 0.01), smoker (*p* = 0.02), and alcohol history (*p* = 0.02). The expression of INMT did not correlate statistically significantly with other clinical-pathological characteristics, including N stage, M stage, clinical stage, age, and radiation therapy. As a result of using the Fisher exact test, as well as the chi-square test (Supplementary Table S1), similar results were obtained. Additional analysis revealed that the AUC value for INMT was 0.703 (CI: 0.620–0.786) (Figure 2K). Furthermore, univariate logistic regression of INMT expression (Table 1) further demonstrated that INMT expression was also closely associated with clinical-characteristics, including histologic grade [Odds Ratio (OR) = 1.660, CI: 1.096–2.530, *p* = 0.017], Gender (OR = 1.637, CI: 1.099–2.451, *p* = 0.016), smoker (OR = 1.874, CI: 1.220–2.906, *p* = 0.004), alcohol history (OR = 1.598, CI: 1.092–2.347, *p* = 0.016), but not T, N, and M stages, clinical stage, age, radiation therapy. At a cutoff of 2.328, INMT had a sensitivity, specificity, and accuracy of 45.5,85.7 and 82.4%, respectively. The negative predictive value was 94.7%, and the positive predictive value was 21.7%. Collectively, these results suggest that INMT may serve as a biomarker for poor prognosis in HNSC.

**TABLE 1:** The association between INMT expression and clinical-pathological characteristics (logistic regression).

Characteristics	Total(N)	Odds Ratio (OR)	*p* value
T stage (T3&T4 vs. T1&T2)	487	0.703 (0.484–1.019)	0.063
N stage (N1&N2&N3 vs. N0)	480	1.016 (0.710–1.454)	0.930
M stage (M1 vs. M0)	477	0.234 (0.012–1.593)	0.195
Clinical stage (Stage III & Stage IV vs. Stage I & Stage II)	488	0.919 (0.603–1.399)	0.695
Histologic grade (G3&G4 vs. G1&G2)	483	1.660 (1.096–2.530)	0.017
Gender (Male vs. Female)	502	1.637 (1.099–2.451)	0.016
Age (>60 vs.≤60)	501	1.041 (0.733–1.478)	0.822
Radiation therapy (Yes vs. No)	441	1.100 (0.743–1.630)	0.634
Smoker (Yes vs. No)	492	1.874 (1.220–2.906)	0.004
Alcohol history (Yes vs. No)	491	1.598 (1.092–2.347)	0.016

### Short overall survival is associated with low mRNA expression of Indiolethylamine-N-methyltransferase

**FIGURE 3:**
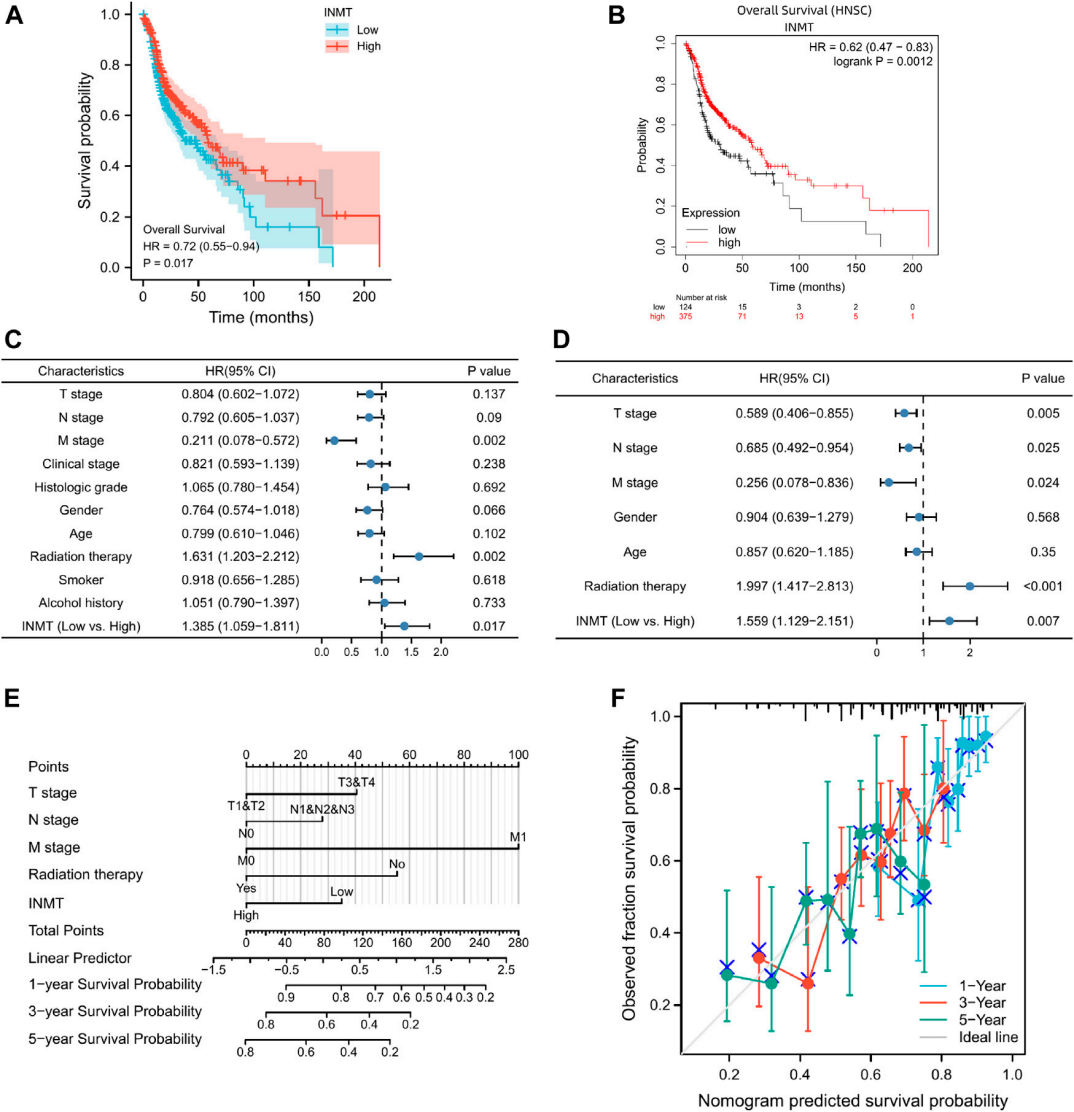
The prognostic significance of INMT expression in HNSC. **(A)** A survival curve for OS based on TCGA data; **(B)** A survival curve for OS based on Kaplan-Meier Plotter; **(C,D)** Univariate and multivariate Cox analyses of INMT and pathological characteristics; **(E)** A nomogram incorporating INMT and other prognostic factors for HNSC utilizing TCGA data; **(F)** The calibration curve of the nomogram.

Kaplan-Meier curves and Kaplan-Meier plots were utilized to investigate the relationship between INMT mRNA expression and the overall survival (OS) of patients with HNSC. Kaplan-Meier survival analysis of the TCGA-HNSC data set uncovered that patients with low INMT expression had a worse overall survival than those with high INMT expression (HR = 0.72, CI: 0.55–0.94, *p* = 0.017; Figure 3A). Similarly, the Kaplan-Meier Plotter result indicated that low INMT expression was related to worse overall survival in HNSC (HR = 0.62, CI: 0.47–0.83, *p* = 0.0002; Figure 3B).

Based on a Cox regression model, univariate analysis of OS identified that poor OS was strongly associated with INMT expression (*p* = 0.017, CI: 1.059–1.81), M stage (*p* = 0.002, CI: 0.078–0.572), radiation therapy (*p* = 0.002, CI: 1.203–2.212) (Supplementry Table S2; Figure 3C). However, at multivariate Cox regression analysis, INMT expression (*p* = 0.007, CI: 1.129–2.151), T stage (*p* = 0.005, CI: 0.406–0.855), N stage (*p* = 0.025, CI: 0.492–0.954), M stage (*p* = 0.024, CI: 0.078–0.836), and radiation therapy (*p* < 0.001, CI: 1.417–2.813) could independently predict adverse OS (Supplementary Table S2; Figure 3D). In addition, this study found that patients with low INMT expression have a 1.559 times greater risk of adverse OS than those with elevated INMT expression (Figure 3D).

Considering the results discussed previously, INMT mRNA may serve as an independent prognostic indicator for HNSC. Based on a multivariate Cox regression analysis of TCGA data, an OS prediction model was developed. We constructed a nomogram of OS that incorporates INMT as well as other prognostic factors, including T stage, N stage, M stage, and radiation therapy (Figure 3E). The higher the point on the nomogram, the worse the prognosis. According to the calibration curve, the performance of INMT was evaluated, and the C-index of the OS was 0.660 (Figure 3F). Overall, this nomogram may be a more accurate predictor of survival than individual prognostic factors for patients with HNSC.

### Functional inference of Indiolethylamine-N-methyltransferase in head and neck squamous cell carcinoma

**FIGURE 4:**
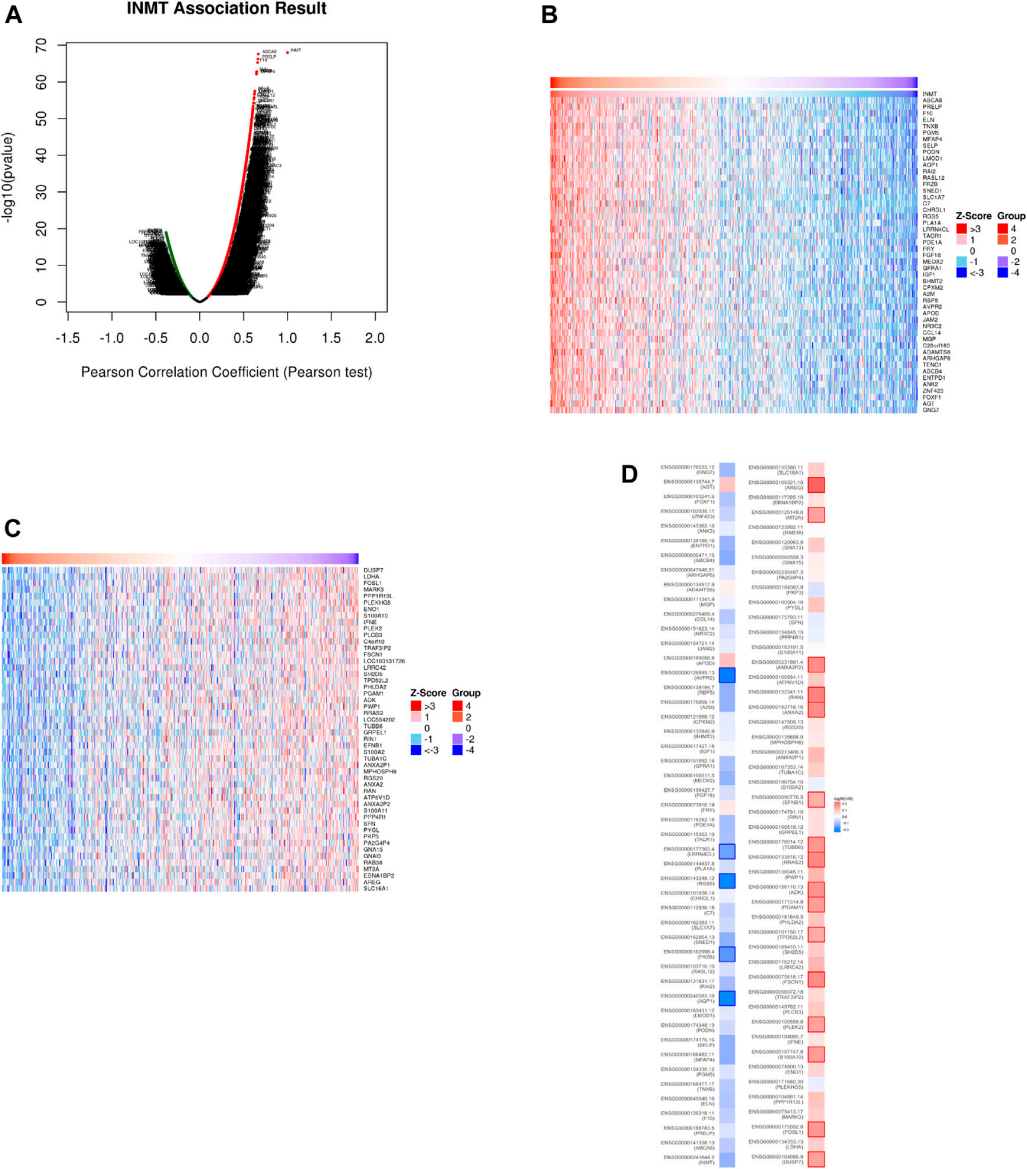
The co-expression genes for INMT in HNSC are derived from the LinkedOmics. **(A)** All the significantly associated genes with INMT were distinguished by the Pearson test in the HNSC cohort. **(B,C)** Heat maps depicting the 50 most significant genes positively and negatively associated with INMT in HNSC. Genes that are positively related are outlined in red, whereas genes that are negatively related are outlined in blue. **(D)** Survival map of the 50 most significant genes positively and negatively related to INMT in HNSC.

In the LinkedOmics web portal, a LinkFinder module was available to investigate the co-expression pattern of INMT in TCGA-HNSC to provide knowledge about INMT’s biological function. As depicted in Figure 4A, 10,950 genes (dark red dots) positively correlate with INMT, whereas 5,433 genes (dark green dots) negatively correlate with INMT. The diagrams in Figures 4B,C illustrate heat maps of the top 50 genes positively and negatively associated with INMT, respectively. In addition, the top 50 genes with a high probability of becoming low-risk markers in HNSC, 43/50 of which had a favorable hazard ratio (HR) should be noted. Contrary to this, we found 40 of the top 50 genes to have unfavorable HR among the top 50 negatively remarkable genes (Figure 4D).

Analysis of the KEGG pathways indicated enrichment in riboflavin metabolism, dilated cardiomyopathy, renin-angiotensin system, hematopoietic cell lineage, cGMP-PKG signaling pathway, Ras signaling pathway, and so on (Supplementary Figure 1A). In addition, GO term annotation revealed that co-expressed genes of INMT were primarily related to organ growth, regulation of metal ion transport, B cell activation, etc., with the biological process (Supplementary Figure 1B), a protein complex involved in cell adhesion, platelet dense granule, sarcoplasm, etc., with the cellular components (Supplementary Figure 1C), and extracellular matrix structural constituent, nucleotide receptor activity, purinergic receptor activity, etc., with the molecular function (Supplementary Figure 1D).

**FIGURE 5:**
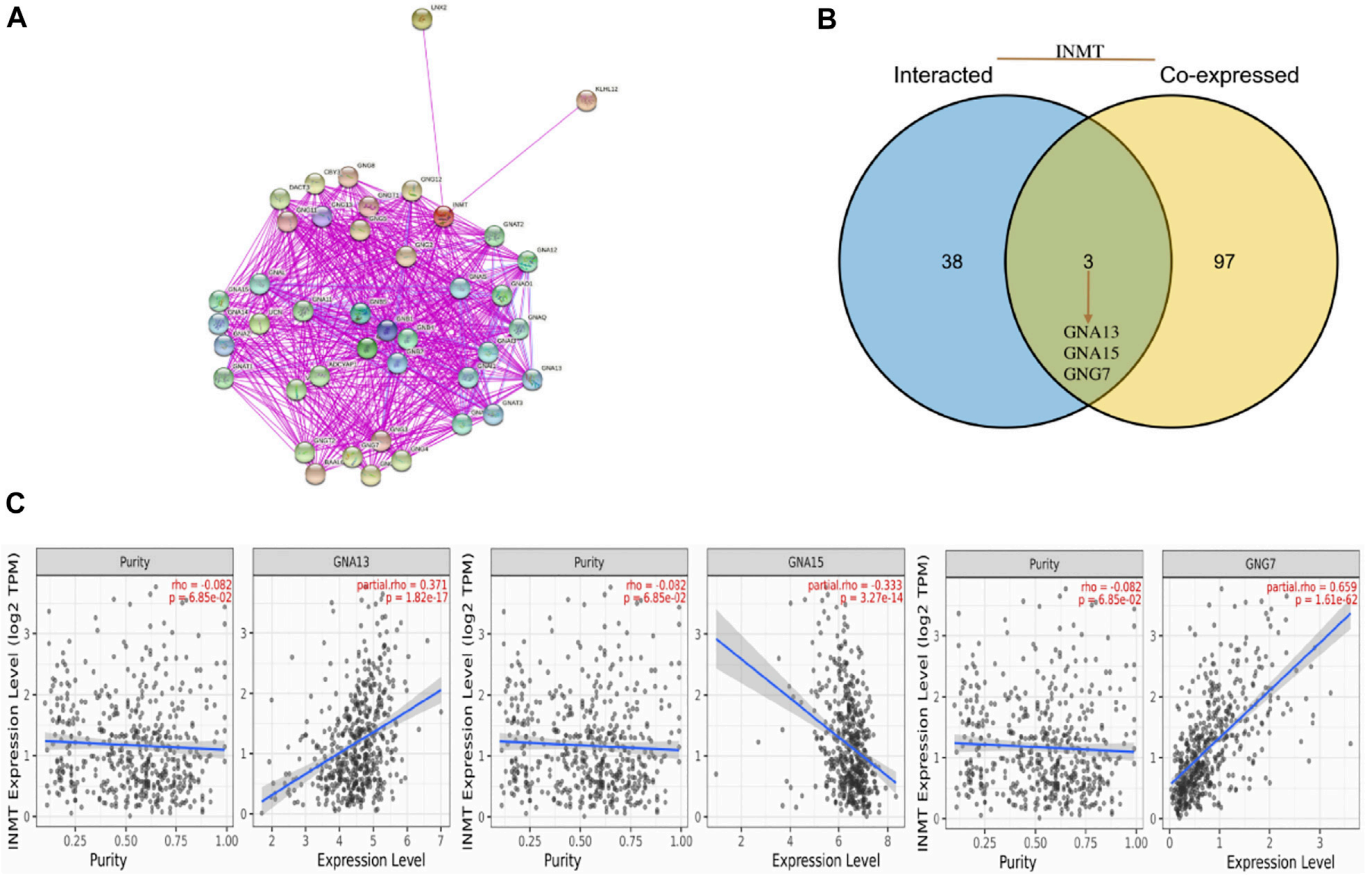
An analysis of INMT-related genes based on PPI networks. **(A)** A network visualization for INMT-binding proteins was created using the STRING database. **(B)** An intersection analysis was performed between co-expressed genes and genes that interacted with INMT. **(C)** INMT expression correlates with screened genes including GNA13, GNA15, and GNG7.

To further explore the internal mechanism underlying the INMT gene’s involvement in tumorigenesis, the STRING website was utilized to explore the PPI network analysis. With the help of experimental evidence, Figure 5A visualized the interaction network of 50 INMT-binding proteins. We also screened out the common genes such as GNA13, GNA15, and GNG7 by comparing the top 50 co-expressed genes with the top 50 interacted genes (Figure 5B). Furthermore, the level of INMT expression was strikingly positively associated with that of GNA13 (*r* = 0.371, *p* = 1.82e-17), GNG7 (*r* = 0.659, *p* = 1.61e−62) and negatively associated with that of GNA15 (*r* = −0.333, *p* = 3.27e−14) (Figure 5C).

### Expression of Indiolethylamine-N-methyltransferase is related to its methylation

**FIGURE 6:**
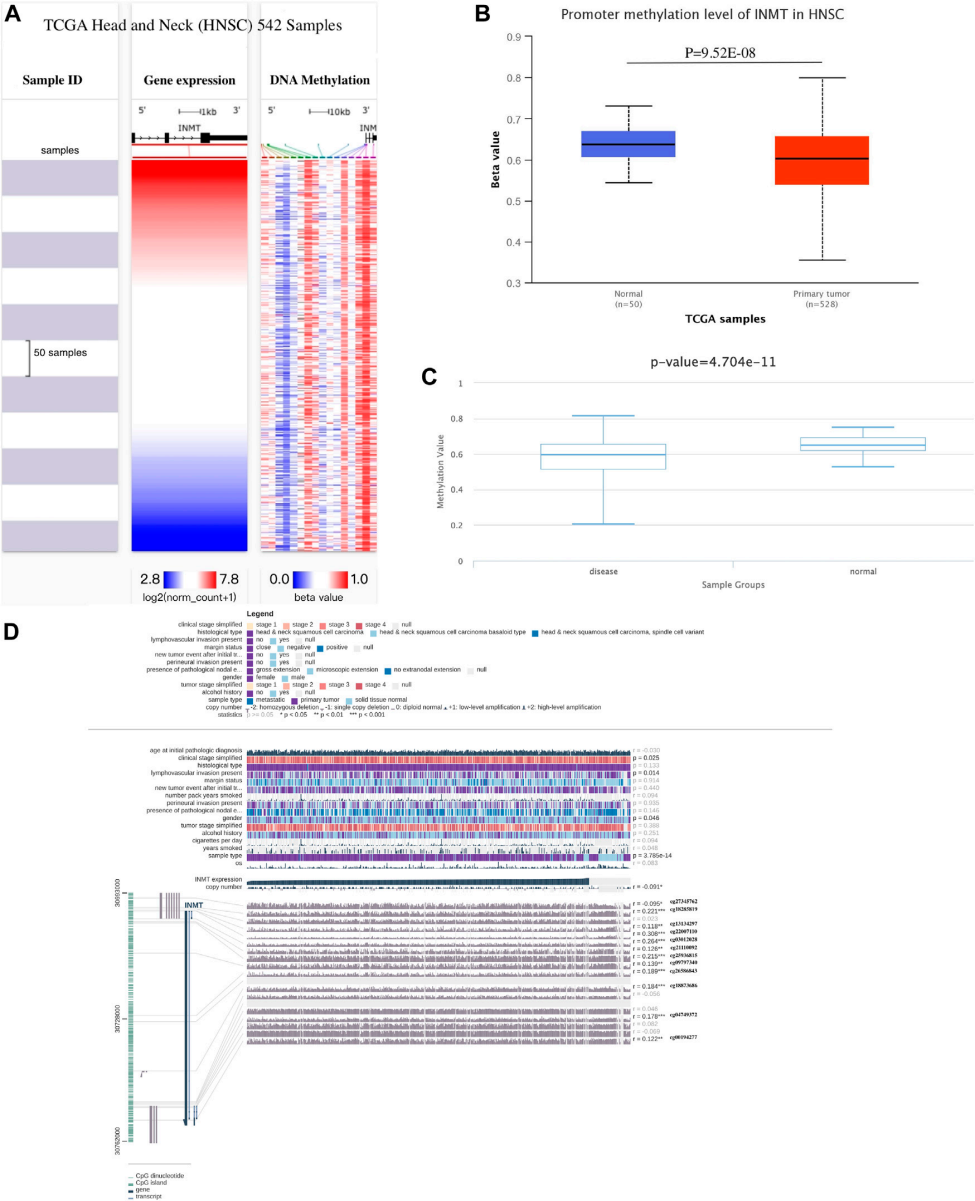
Methylation analysis of INMT. **(A)** Heatmap illustrating the correlations between INMT mRNA and methylation in HNSC as determined by UCSC Xena. **(B)** Methylation was assessed *via* UALCAN. **(C)** DiseaseMeth version 2.0 was used to determine methylation. **(D)** Methylation of the INMT DNA sequence related to gene expression was visualized utilizing MEXPRESS. A blue line in the plot illustrates the expression of INMT. On the right, you can see Pearson’s coefficients of correlation and *p* values for methylation sites and query gene expression.

Various methods were used to investigate the correlation between INMT expression levels and their methylation status to elucidate the abnormal downregulation mechanisms found in HNSC tissues. Using the UCSC Xena database, we first examined the DNA methylation levels of the INMT in HNSC. INMT mRNA expression is related to DNA methylation (Figure 6A). As shown in Figure 6B, the results of the UALCAN analysis indicated that INMT had a trend of higher methylation levels in normal head and neck samples than in HNSC samples (*p* = 9.52E-08). As in the case of DiseaseMeth version 2.0, the methylation of INMT in paracancerous normal tissues was greater than that in HNSC tissues (*p* = 4.70E-11; Figure 6C). Moreover, through MEXPRESS database, we identified eleven methylation sites (cg18285819, cg13134297, cg22007110, cg03012028, cg21110092, cg25936815, cg09797340, cg26586843, cg18873686, cg04749372, cg00194277) in the DNA sequences of INMT that were positively related to their expression levels. Conversely, only one methylation site (cg27345762) was negatively correlated with INMT expression levels (Figure 6D). Third, we presented heatmaps of the differentially methylated regions associated with INMT (Supplementary Figure 2A). Interestingly, we were able to validate the two predicted methylation sites (cg04749372 and cg00194277) using the Methsurv database. cg04749372 was detected in the open sea region and 1stExon region, and cg00194277 was detected in the open sea region and 3′ UTR region (Supplementary Figure 2A). As we continued to use Methsurv, we found that cg18589624, located in the TSS1500 region and open ocean, was associated with a poor prognosis (Supplementary Figure 2B).

### Association between Indiolethylamine-N-methyltransferase with immune infiltration level

**FIGURE 7:**
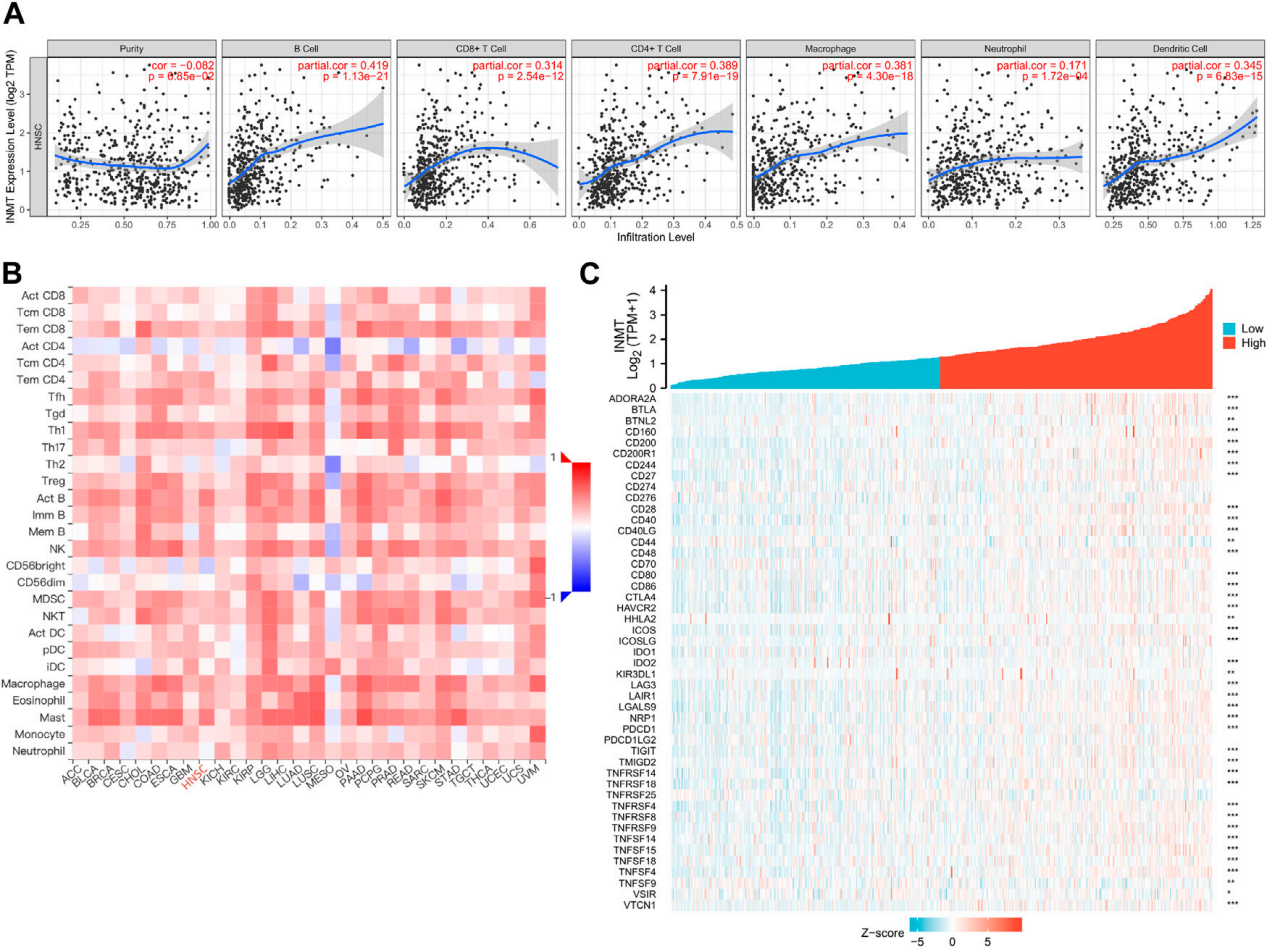
The relationship between the INMT level and immune infiltration level in HNSC. **(A)** Analysis of the correlation between INMT expression and the levels of immune cell infiltration in HNSC tissues using the TIMER database. **(B)** Relationship between expression of INMT and 28 types of TILs across human heterogeneous cancers. **(C)** Relationship between INMT expression and the gene levels of more than 40 common immune checkpoints in HNSC.

As both KEGG and GO enrichment analyses illustrated that INMT may be involved in the tumor immune response, employing the TIMER, we first investigated the association between INMT expression and the six types of tumor-infiltrating immune cells. Pearson correlation analysis (Figure 7A) confirmed that there were significant positive associations between INMT expression and all six types of immune cells. Additionally, we utilized ssGSEA to assess the interrelation between INMT and 24 immune cell subsets in HNSC and found that INMT is strongly correlated with B cells, CD8+T cells, Cytotoxic cells, DC, Eosinophils, iDC, Macrophages, Mast cells, NK CD56bright cells, NK cells, pDC, T cells, T helper cells, Tem, TFH, Th1 cells, Th17 cells, Th2 cells, TReg (Supplementary Figure 3A). INMT exhibits a close negative correlation with Tgd (Supplementry Figure 3A). As well, similar results were also achieved using the TISIDB database (Figure 7B; Table 2). The TISIDB database was also examined to determine the interrelation between INMT methylation and 28 types of tumor-infiltrating lymphocytes (TILs). The data shown in Supplementary Figure 3B and Table 2 show that INMT methylation was significantly positively related to 27 kinds of immune cells, except for CD56dim NKT cells. We then examined how INMT expression correlates with tumor-infiltrating immune cell gene marker levels in HNSC samples by examining the TIMER website. As shown in Table 3, INMT levels in HNSC tissues were strongly associated with fourteen immune cells’ all markers (B cells, CD8^+^ T cells, dendritic cells, M2 macrophages, monocytes, neutrophils, T general cells, T exhaustion cells, TAMs, Tfhs, Th1s, Th2s, Th17s, and Tregs).

**TABLE 2:** Tumor lymphocyte infiltration in HNSC is related to INMT expression and methylation, respectively (TISIDB).

	INMT expression		INMT methylation	
	Rho	*P*	Rho	*P*
Activated CD8 T cell (Act_CD8)	0.096	0.028	0.356	3.78E-17
Central memory CD8 T cell (Tcm _CD8)	0.111	0.011	0.213	1.02E-06
Effector memory CD8 T cell (Tem _CD8)	0.187	1.71E-05	0.423	<2.2E-16
Activated CD4 T cell (Act_CD4)	0.123	0.005	0.31	5.67E-13
Central memory CD4 T cell (Tcm _CD4)	−0.065	0.137	0.187	1.83E-05
Effector memory CD4 T cell (Tem _CD4)	0.359	1.48E-17	0.21	1.37E-06
T follicular helper cell (Tfh)	0.309	6.49E-13	0.343	9.46E-16
Gamma delta T cell (Tgd)	0.029	0.515	0.332	9.44E-15
Type 1 T helper cell (Th1)	0.371	<2.2E-16	0.351	1.64E-16
Type 17 T helper cell (Th17)	0.183	2.78E-05	0.24	3.04E-08
Type 2 T helper cell (Th2)	0.135	0.002	0.296	7.07E-12
Regulatory T cell (Treg)	0.229	1.25E-07	0.357	2.92E-17
Activated B cell (Act_B)	0.48	<2.2E-16	0.237	4.53E-08
Immature B cell (Imm_B)	0.348	3.13E-16	0.372	<2.2E-16
Memory B cell (Mem_B)	0.361	7.23E-18	0.107	0.015
natural killer cell (NK)	0.365	6.56E-19	0.252	6.09E-09
CD56bright natural killer cell (CD56bright)	−0.195	7.81E-06	0.211	1.23E-06
CD56dim natural killer cell (CD56dim)	0.005	0.907	−0.014	0.745
Myeloid derived suppressor cell (MDSC)	0.228	1.43E-07	0.433	<2.2E-16
Natural killer T cell (NKT)	0.242	2.25E-08	0.295	7.08E-12
Activated dendritic cell (Act_DC)	0.026	0.552	0.3	3.32E-12
Plasmacytoid dendritic cell (pDC)	0.237	4.77E-08	0.144	0.001
Immature dendritic cell (iDC)	0.026	0.561	0.237	4.69E-08
Macrophage (Macrophage)	0.367	<2.2E-16	0.266	7.85E-10
Eosinophi (Eosinophil)	0.332	9.54E-15	0.234	7.17E-08
Mast (Mast)	0.495	<2.2E-16	0.161	<0.001
Monocyte (Monocyte)	0.084	0.056	0.33	1.28E-14
Neutrophil (Neutroph)	−0.035	0.428	0.24	3.07E-08

**TABLE 3:** The relevance between INMT and the biomarkers of immune cells in HNSC was analyzed utilizing the Timer platform.

Description	Gene markers	HNSC			
		None		Purity	
		cor	*p*	cor	*p*
B cell	CD19	0.476	***	0.476	***
	CD79A	0.522	***	0.526	***
CD8^+^ T cell	CD8A	0.27	***	0.249	***
	CD8B	0.324	***	0.305	***
Dendritic cell	ITGAX	0.388	***	0.376	***
	NRP1	0.307	***	0.293	***
	CD1C	0.427	***	0.416	***
	HLA-DPA1	0.313	***	0.294	***
	HLA-DRA	0.304	***	0.287	***
	HLA-DQB1	0.245	***	0.231	***
	HLA-DPB1	0.337	***	0.32	***
M1 Macrophage	PTGS2	0.01	0.82	0.025	0.577
	IRF5	0.255	***	0.251	***
	NOS2	0.39	***	0.405	***
M2 Macrophage	MS4A4A	0.342	***	0.318	***
	VSIG4	0.321	***	0.302	***
	CD163	0.331	***	0.313	***
Monocyte	CSF1R	0.399	***	0.383	***
	CD86	0.285	***	0.266	***
Natural killer cell	KIR2DS4	0.062	0.159	0.044	0.331
	KIR3DL3	0.094	0.032	0.073	0.108
	KIR3DL2	0.292	***	0.279	***
	KIR3DL1	0.159	**	0.146	*
	KIR2DL4	0.086	0.049	0.091	0.045
	KIR2DL3	0.121	*	0.102	0.023
	KIR2DL1	0.068	0.123	0.056	0.212
Neutrophils	CCR7	0.489	***	0.493	***
	ITGAM	0.399	***	0.378	***
	CEACAM8	0.12	*	0.108	0.017
T cell (general)	CD3D	0.326	***	0.313	***
	CD3E	0.393	***	0.383	***
	CD2	0.39	***	0.379	***
T cell exhaustion	CTLA4	0.309	***	0.298	***
	LAG3	0.187	***	0.173	**
	HAVCR2	0.326	***	0.306	***
	GZMB	0.154	**	0.135	*
	PDCD1	0.3	***	0.286	***
TAM	CCL2	0.5	***	0.484	***
	IL10	0.406	***	0.395	***
	CD68	0.177	***	0.157	**
Tfh	BCL6	0.359	***	0.378	***
	IL21	0.294	***	0.264	***
Th1	TBX21	0.313	***	0.297	***
	STAT4	0.324	***	0.314	***
	IFNG	0.12	*	0.095	0.035
	IL13	0.201	***	0.183	***
Th2	GATA3	0.246	***	0.236	***
	STAT6	0.157	**	0.162	**
	STAT5A	0.378	***	0.355	***
Th17	STAT3	0.298	***	0.29	***
	IL17A	0.205	***	0.196	***
Treg	FOXP3	0.454	***	0.448	***
	CCR8	0.454	***	0.443	***
	STAT5B	0.442	***	0.429	***
	TGFB1	−0.089	0.041	−0.088	0.052

*p< 0.01; **p< 0.001; ***p< 0.0001.

Immune checkpoint inhibitors (ICIs), a novel approach to cancer immunotherapy, have already been shown to improve the outcomes of many types of cancer patients (Pardoll, 2012; Topalian et al., 2015). We then investigated the association between the expression of the INMT gene and the expression of over 40 common immune-control genes. A noteworthy finding was that INMT expression is related to almost 41 immune checkpoint markers in HNSC, including PDCD1, CTLA4, CD160, CD200, and so on (Figure 7C). The data presented here indicate that PD-1 (PDCD1) and CTLA4, two biomarkers used to evaluate immune-checkpoint inhibitor efficacy (Pardoll, 2012), showed a highly significant correlation with INMT expression in HNSC.

The last analysis is performed in HNSC using Kaplan-Meier plotters to explore the association between INMT expression and the prognosis of eight immune cells. In addition, we identified that patients with low INMT levels in enriched B cells (*p* = 0.0019), CD4^+^ memory T cells (*p* = 0.0036), CD8^+^ T cells (*p* = 4.3e-05), macrophages (*p* = 0.015), regulatory T-cells (*p* = 0.0039), type 1 T-helper cells (*p* = 0.0014), type 2 T-helper cells (*p* = 0.001) had a worse prognosis (Supplementary Figure 4). Hence, these findings strongly demonstrate that the INMT gene might contribute to tumor immunity.

## Discussion

Even though significant progress over the past few years, there is continued evidence of increased morbidity and mortality associated with HNSC. To improve the survival rate of HNSC patients, it is imperative to make an accurate prediction of prognosis. Thus, HNSC needs useful therapeutic targets or the identification of potential biomarkers of prognosis. The purpose of this article is to examine the role of INMT as a potential marker in HNSC as well as its potential prognostic value.

INMT belongs to a large class of N-methyltransferases that utilize SAM as a methyl donor. SAM is used by INMT to transfer methyl groups to the nitrogen of substrates containing indolyl alkyl amino groups and, subsequently, to create SAH. During the past decade, the antiproliferative, proapoptotic, and antimetastatic properties of SAM have been extensively studied in pan-cancer. SAM has been demonstrated to induce cell cycle arrest, inhibit the migration and invasion of two HNSC cell lines (oral Cal-33 and laryngeal JHU-SCC-011), and modulate through the main signaling pathways such as AKT, β-catenin, and SMAD (Mosca et al., 2020). According to the Gene Cards database (Safran et al., 2021), NNMT is an important paralog of INMT. Expression of NNMT on ovarian cancer cells supported migration, proliferation, growth, and metastasis *in vivo*. In cancer-associated fibroblasts (CAFs), the expression of NNMT was associated with depletion of SAM and a reduction in histone methylation, resulting in alterations in gene expression (Eckert et al., 2019).

Evidence suggests that the expression of INMT is reduced in several cancers (Kopantzev et al., 2008; Larkin et al., 2012; Schulten et al., 2016). As a result of pan-cancer analysis, we also discovered that different expressions of INMT were observed in various types of tumors (Figure 1A). Research has shown that when INMT is overexpressed in prostate cancer cells, INMT inhibits cell proliferation and induces apoptosis by activating MAPK, TGFβ, and Wnt signaling pathways (Jianfeng et al., 2022).

Another study examined the role of PTEN in endometrial cancer and found that deregulation of the INMT gene is linked to the absence of PTEN (Lian et al., 2006). The current study found that INMT is downregulated in HNSC at both the mRNA level and the protein level (Figures 1C–E). Taken together, we hypothesized that INMT may also act a crucial part in the initiation, progression, and metastatic phases of HNSC.

There is a correlation between reduced INMT expression and several clinical parameters including T stage, histologic grade, gender, smoking status, and alcohol consumption (Figure 2) as well as poor overall survival (Figures 3A,B). In addition, the multivariate analysis further indicates that INMT expression was an independent predictor of prognosis in HNSC patients (Figures 3C–F; Table 1; Supplementary Tables S1, S2). Hence, INMT downregulation occurs in nearly all HNSC samples, contributing to their progression. In terms of its potential as a prognostic marker, INMT warrants further clinical investigation.

To discover more about the role of INMT in HNSC, path enrichment analysis was carried out using the LinkedOmics database. The enrichment analysis revealed that low INMT expression was enriched in pathways and biological functions that were related to tumorigenesis, such as the Ras, cGMP-PKG signaling pathways, and so on (Figure 4). Cellular receptors, such as RTKs and GPCRs, activate classic Ras signaling (Rauen, 2013). It is known that Ras-GTP stimulates a wide range of downstream effectors, although the best known of these are the MAPK, the PI3K (Rodriguez-Viciana et al., 1994; Pacold et al., 2000), and the Ral pathways (Rebhun et al., 2000). The PI3K/AKT and Raf/MAPK/ERK pathways are frequently mutated in cancer, resulting in aberrant activation of signaling pathways (Yang et al., 2019; Guo et al., 2020). Cancer cells, particularly those of the breast and colon, have been identified to be susceptible to the cyclic GMP (cGMP)/protein kinase G (PKG) cascade (Browning et al., 2010; Fallahian et al., 2011; Wen et al., 2015). The HNSC cells also express essential components of the cGMP-PKG signaling axis (Tuttle et al., 2016).

We screened out the common members of INMT, such as GNA13, GNA15, and GNG7, by comparing INMT-top50_co-expressed genes with INMT-interacted genes (Figure 5B). According to a recent study, GNA13 expression is associated with drug resistance and tumor-initiating phenotypes in HNSC (Rasheed et al., 2018). In addition, recurrent DNA hypermethylation and reduced protein expression in the GNG7 gene have been reported in HNSC (Hartmann et al., 2012).

CpGs methylation in promoter regions is usually regarded as a repressive mark because it inhibits gene expression. In response, we looked online database for DNA methylation patterns that might explain INMT’s downregulation in HNSC. In comparison with adjacent normal samples, HNSC samples exhibited hypomethylation of INMT. Recent studies suggest, however, that they can also act as activation marks, depending on their location and density along gene structures (Jones, 2012; Yang et al., 2014), as methylation of gene bodies and CpG-poor sites has been observed in active genes (Shenker and Flanagan, 2012). Although the mechanism underlying transcription elongation remains unclear, it appears to be related to structural requirements (Jones, 2012). By analyzing 542 human transcription factors (TFs) with methylation-sensitive SELEX (systematic evolution of ligands by exponential enrichment), a paper on the impact of cytosine methylation on DNA binding specificities published in Science in 2017 (Yin et al., 2017), they discovered that numerous TFs favor CpG-methylated regions. The majority of these belong to the extended family of homeodomains. Based on structural analysis, methylcytosine specificity depends on hydrophobic interactions with the 5-methyl group of methylcytosine. Combined with this paper and by querying the Gene Cards database, we speculated that the promoter methylation of INMT in HNSCC can positively regulate the expression of INMT, which may be related to the binding of transcription factors such as SCRT2 and NR2F1.

Tumor-infiltrating lymphocytes (TILs) are stromal cells that are capable of enhancing and maintaining an immunosuppressive microenvironment, stimulating immune escape, and consequently promoting tumor progression (Callahan and Wolchok, 2019; Demaria and Vivier, 2020; Yu et al., 2020). In the Golgi apparatus and vesicles, INMT is mainly involved in protein processing and cellular secretion. Through cell secretion, tumor cells can alter the tumor microenvironment. Tissue immunotherapy is critically impacted by the complexity and diversity of immune cell infiltration in the tumor microenvironment (Jianfeng et al., 2022). Our study revealed that INMT is closely linked to the tumor microenvironment, as an enzyme crucial to tryptophan metabolism. Upon comprehensive analysis of the results obtained in Timer, ssGSEA, and TISIDB, it can be seen that INMT expression was positively correlated with the infiltration of B cells, CD8 + T cells, Eosinophils, Macrophages, Mast cells, NK cells, pDC, T cells, T helper cells, Tem, Tfh, Th1 cells, Th17 cells, Th2 cells, and Treg (Figures 7A,B; Supplementary Figure 3A; Table 2). After correction for cell purity, INMT showed a positive interrelation with the majority of immune cell markers (Table 3). The findings of this study suggest that INMT is associated with the immune infiltration of HNSC. Specifically, the INMT level was significantly correlated with several markers of T helper cells (Th1, Th2, Tfh, and Th17) in HNSC. Consequently, it may have contributed to the poor prognosis of HNSC through the recruitment and regulation of immune cells. In addition, Mutations of p53 causing hotspots are often immunogenic, eliciting intratumoral T cell responses. INMT and p53 can be combined to form targeted anticancer immunotherapies (Chasov et al., 2021).

In summary, we demonstrated for the first time that downregulated INMT is strongly associated with clinicopathological characteristics, poor prognoses, varied pathways, DNA methylation, and immune cell infiltration in HNSC. As a result, this study provides valuable insights into further research on tumor therapy in HNSC. This research is part of a larger project, which will include validation in a prospectively enrolled study population.

## Data Availability

The original contributions presented in the study are included in the article/Supplementary Material, further inquiries can be directed to the corresponding author.
