# Supplementary material for: Poor prognosis, hypomethylation, and immune infiltrates are associated with downregulation of INMT in head and neck squamous cell carcinoma
Source: Front Genet. 2022 Sep 15;13:917344. doi: 10.3389/fgene.2022.917344 (PMC9520724; doi:10.3389/fgene.2022.917344)
Supplement: Supplementary file 1 [file Presentation1.pdf]

Supplemental Figures

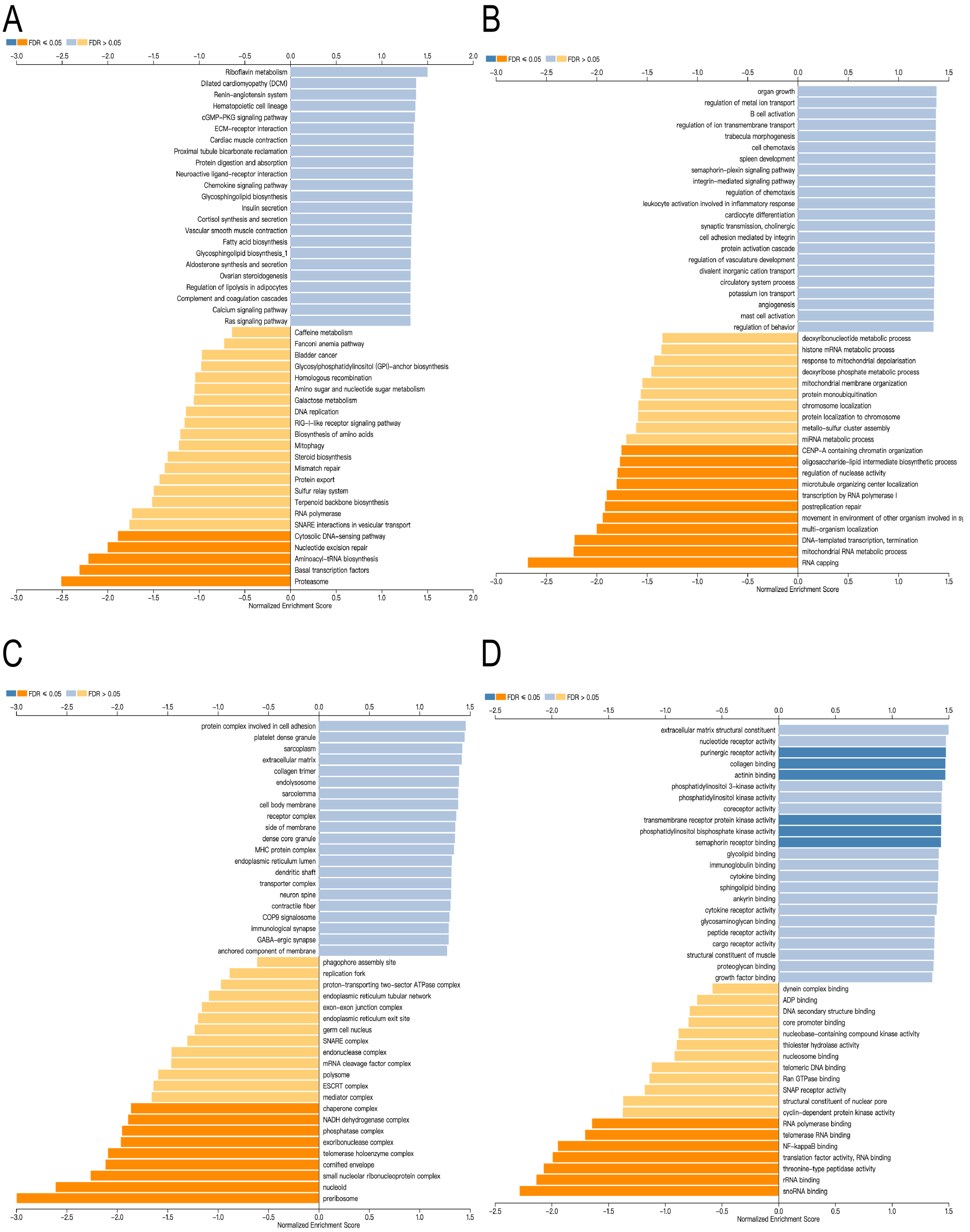

Supplemental Figure 1

The co-expression genes for INMT in HNSC are derived from the LinkedOmics. (A-D) KEGG pathways and GO annotations of INMT in HNSC cohort.

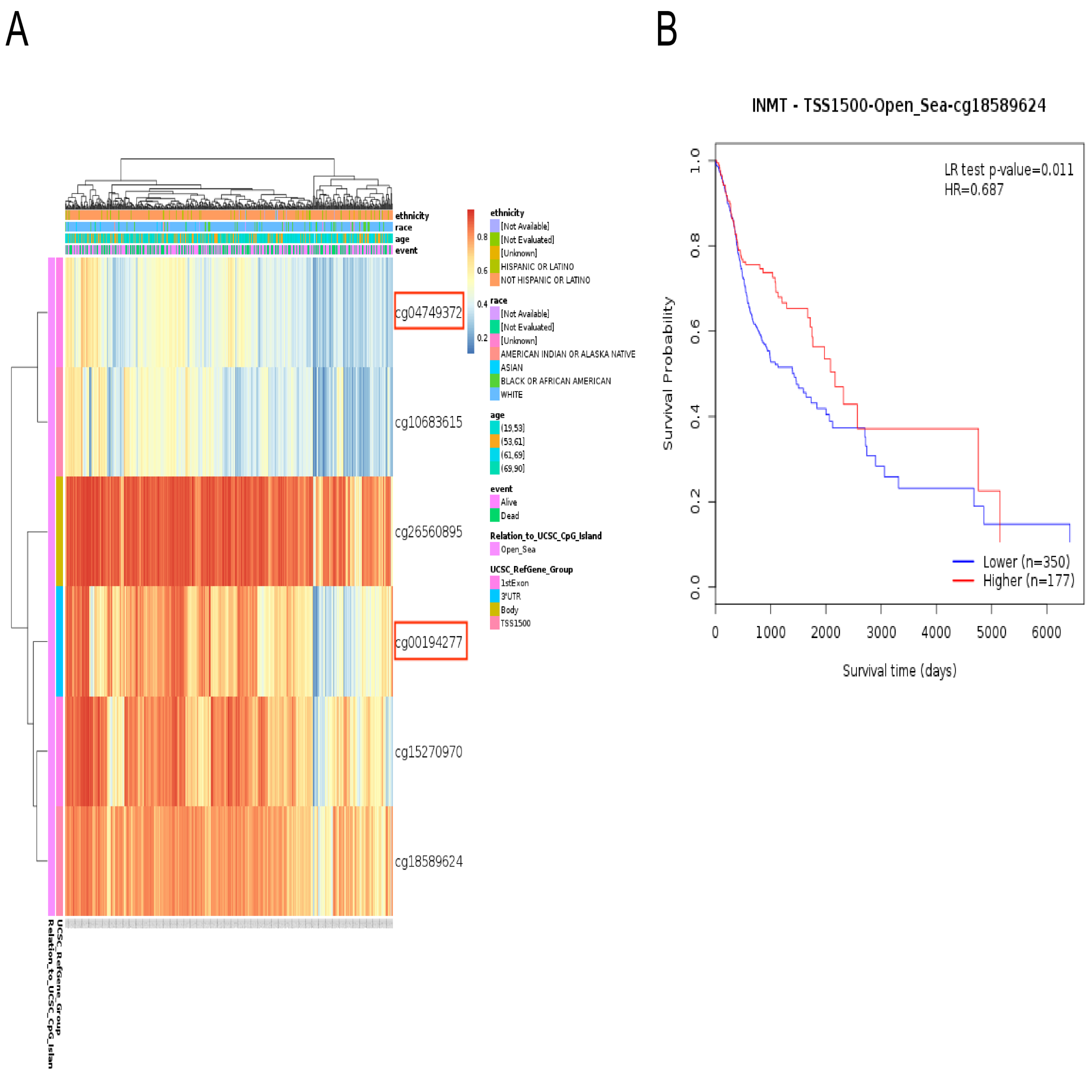

Supplemental Figure 2

Methylation analysis of INMT

(A-B) The visualization of the correlation between methylation levels and INMT expression as well as the Kaplan-Meier survival of the promoter of INMT via Methsurv.

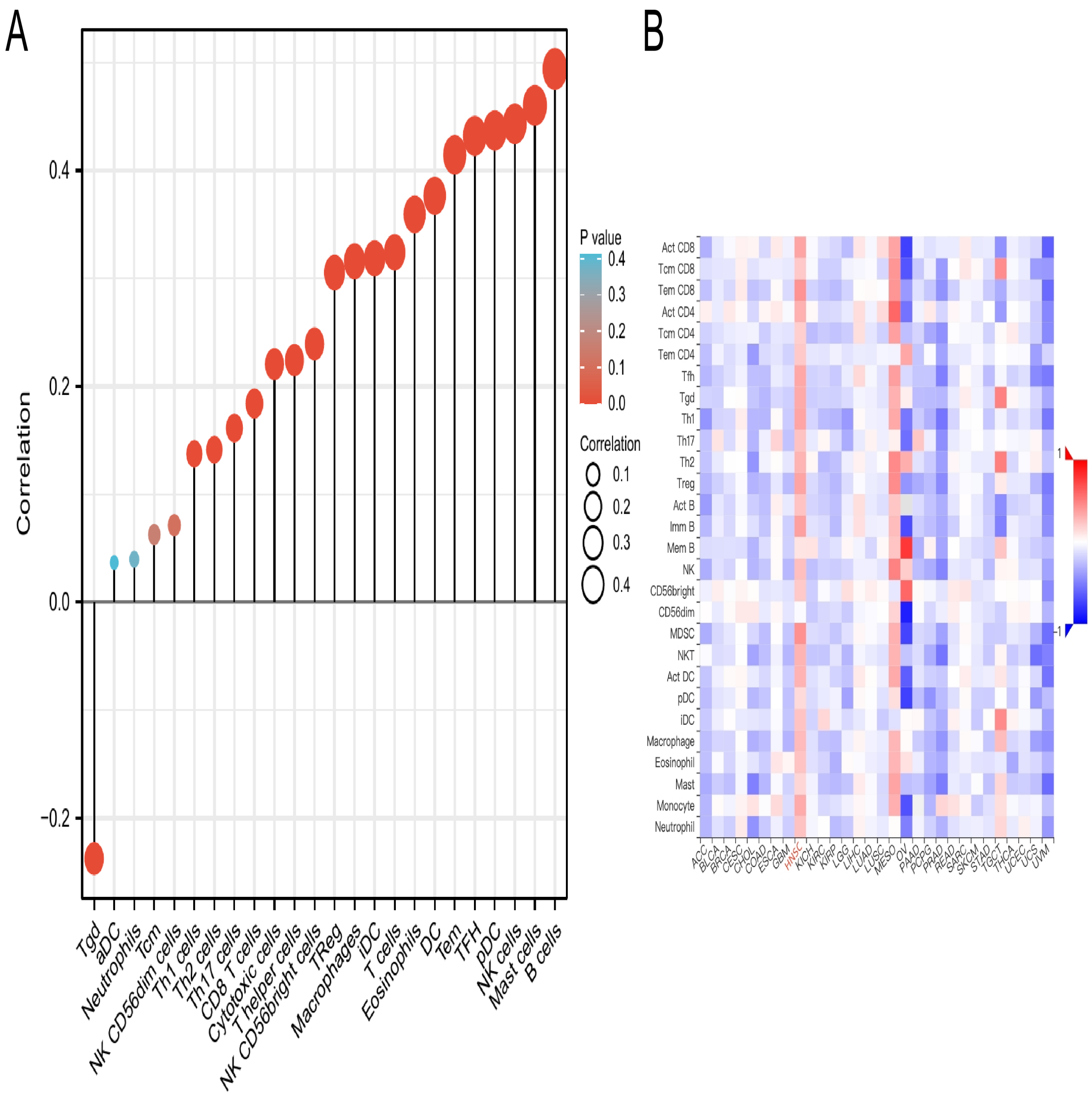

Supplemental Figure 3

Methylation analysis of INMT

(A) Analysis of the correlation between INMT expression and the levels of immune cell infiltration in HNSC tissues using the ssGSEA analysis. (B) Relationship between methylation of INMT and 28 types of TILs across human heterogeneous cancers.

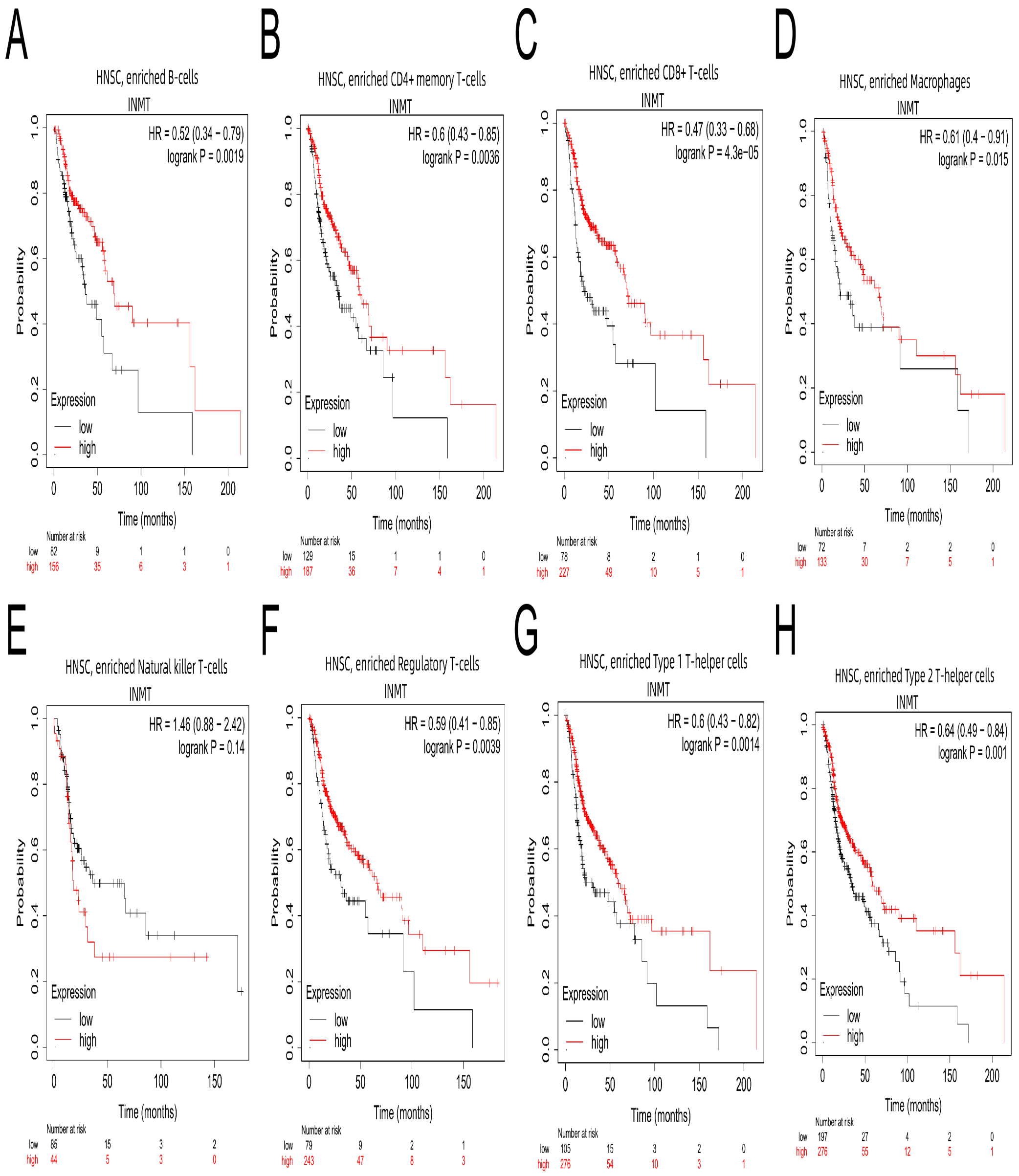

Supplemental Figure 4

Based on subgroups of immune cells, comparison of Kaplan-Meier survival curves for HNSC expressing high or low INMT levels. (A–H) Low INMT level enriched in B cells, CD4+ memory T cells, CD8+ T cells, macrophages, Regulatory T cells, Th1 cells and Th2 cells had worse OS in HNSC.

Supplemental Tables

Supplemental Table 1

Clinical characteristics of the HNSC patients (TCGA).

Characteristic	Total	Low expression of INMT	High expression of INMT	P-value
	N (%)	N (%)	N (%)	
**T stage**				0.203
T1	33 (6.8%)	12 (2.5%)	21 (4.3%)	
T2	144 (29.5%)	65 (13.3%)	79 (16.2%)	
T3	131 (26.9%)	66 (13.6%)	65 (13.3%)	
T4	179 (36.7%)	96 (19.7%)	83 (17%)	
**N stage**				0.505
N0	239 (49.8%)	116 (24.2%)	123 (25.6%)	
N1	80 (16.7%)	44 (9.2%)	36 (7.5%)	
N2	154 (32.1%)	69 (14.4%)	85 (17.7%)	
N3	7 (1.4%)	3 (0.6%)	4 (0.8%)	
**M stage**				0.205
M0	472 (99.0%)	228 (47.8%)	244 (51.2%)	
M1	5 (1.0%)	4 (0.8%)	1 (0.2%)	
**Clinical stage**				0.559
Stage I	19 (3.9%)	8 (1.6%)	11 (2.3%)	
Stage II	95 (19.4%)	46 (9.4%)	49 (10%)	
Stage III	102 (20.9%)	56 (11.5%)	46 (9.4%)	
Stage IV	272 (55.7%)	129 (26.4%)	143 (29.3%)	
**Histologic grade**				< 0.001
G1	62 (12.8%)	44 (9.1%)	18 (3.7%)	
G2	300 (62.1%)	148 (30.6%)	152 (31.5%)	
G3	119 (24.6%)	48 (9.9%)	71 (14.7%)	
G4	2 (0.4%)	1 (0.2%)	1 (0.2%)	
**Gender**				0.020
Female	134 (26.7%)	79 (15.7%)	55 (11%)	
Male	368 (73.3%)	172 (34.3%)	196 (39%)	
**Age**				0.893
<=60	245 (49.0%)	124 (24.8%)	121 (24.2%)	
>60	256 (51.0%)	127 (25.3%)	129 (25.7%)	
**Smoker**				0.006
No	111 (22.5%)	69 (14%)	42 (8.5%)	
Yes	381 (77.5%)	178 (36.2%)	203 (41.3%)	
**Alcohol history**				0.021
No	158 (32.1%)	91 (18.5%)	67 (13.6%)	

Yes	333 (67.9%)	153 (31.2%)	180 (36.7%)	
**Radiation therapy**				0.707
No	154 (34.9%）	82 (18.6%)	72 (16.3%)	
Yes	287 (65.1%)	146 (33.1%)	141 (32%)	
**Primary therapy outcome**				< 0.001
PD	41 (9.8%）	30 (7.2%)	11 (2.6%)	
SD	6 (1.4%)	1 (0.2%)	5 (1.2%)	
PR	6 (1.4%）	5 (1.2%)	1 (0.2%)	
CR	365 (87.3%)	174 (41.6%)	191 (45.7%)	
Age, meidan (IQR)		61 (53, 69)	61 (54, 68)	0.823

Supplemental Table 2

Univariate regression and multivariate survival method (Overall Survival) of prognostic covariates in patients with HNSC

Characteristics	Total(N)	HR (95% CI) Univariate analysis	P value Univariate analysis	HR (95% CI) Multivariate analysis	P value Multivariate analysis
T stage (T1&T2 vs. T3&T4)	486	0.804 (0.602-1.072)	0.137	0.589 (0.406-0.855)	0.005
N stage (N0 vs. N1&N2&N3)	479	0.792 (0.605-1.037)	0.090	0.685 (0.492-0.954)	0.025
M stage (M0 vs. M1)	476	0.211 (0.078-0.572)	0.002	0.256 (0.078-0.836)	0.024
Clinical stage (Stage I & Stage II vs. Stage III & Stage IV)	487	0.821 (0.593-1.139)	0.238		
Histologic grade (G1&G2 vs. G3&G4)	482	1.065 (0.780-1.454)	0.692		
Gender (Male vs. Female)	501	0.764 (0.574-1.018)	0.066	0.904 (0.639-1.279)	0.568
Age (<=60vs.>60)	501	0.799 (0.610-1.046)	0.102	0.857 (0.620-1.185)	0.350
Radiation therapy (No vs. Yes)	440	1.631 (1.203-2.212)	0.002	1.997 (1.417-2.813)	<0.001
Smoker (No vs. Yes)	491	0.918 (0.656-1.285)	0.618		
Alcohol history (No vs. Yes)	490	1.051 (0.790-1.397)	0.733		
INMT (Low vs. High)	501	1.385 (1.059-1.811)	0.017	1.559 (1.129-2.151)	0.007
